# The effects of graded levels of calorie restriction: VII. Topological rearrangement of hypothalamic aging networks

**DOI:** 10.18632/aging.100944

**Published:** 2016-04-23

**Authors:** Davina Derous, Sharon E. Mitchell, Cara L. Green, Yingchun Wang, Jing Dong J. Han, Luonan Chen, Daniel E.L. Promislow, David Lusseau, John R. Speakman, Alex Douglas

**Affiliations:** ^1^ Institute of Biological and Environmental Sciences, University of Aberdeen, Aberdeen, Scotland, AB24 2TZ, UK; ^2^ Centre for Genome Enabled Biology and Medicine, University of Aberdeen, Aberdeen, Scotland, AB24 3RL, UK; ^3^ State Key Laboratory of Molecular Developmental Biology, Institute of Genetics and Developmental Biology, Chinese Academy of Sciences, Chaoyang, Beijing, 100101, China; ^4^ Chinese Academy of Sciences Key Laboratory of Computational Biology, Chinese Academy of Sciences-Max Planck Partner Institute for Computational Biology, Shanghai Institutes for Biological Sciences, Chinese Academy of Sciences, Shanghai, 200031, China; ^5^ Key Laboratory of Systems Biology, Innovation Center for Cell Signaling Network, Institute of Biochemistry and Cell Biology, Shanghai Institute of Biological Sciences, Chinese Academy of Sciences, Shanghai, 200031, China; ^6^ Department of Pathology and Department of Biology, University of Washington, Seattle, WA 98195, USA

**Keywords:** aging, calorie restriction, conditional mutual information, network biology, transcriptomics

## Abstract

Connectivity in a gene-gene network declines with age, typically within gene clusters. We explored the effect of short-term (3 months) graded calorie restriction (CR) (up to 40 %) on network structure of aging-associated genes in the murine hypothalamus by using conditional mutual information. The networks showed a topological rearrangement when exposed to graded CR with a higher relative within cluster connectivity at 40CR. We observed changes in gene centrality concordant with changes in CR level, with *Ppargc1a*, and *Ppt1* having increased centrality and *Etfdh*, *Traf3* and *Abcc1* decreased centrality as CR increased. This change in gene centrality in a graded manner with CR, occurred in the absence of parallel changes in gene expression levels. This study emphasizes the importance of augmenting traditional differential gene expression analyses to better understand structural changes in the transcriptome. Overall our results suggested that CR induced changes in centrality of biological relevant genes that play an important role in preventing the age-associated loss of network integrity irrespective of their gene expression levels.

## INTRODUCTION

Brain aging has an impact on cognitive function and forms a major risk for the development of neuro-degenerative disorders such as Parkinson's, Huntington's and Alzheimer's disease [1]. In murine brain tissue, aging is associated with transcriptional changes in genes that induce an elevated inflammatory response and increased oxidative stress [2,3]. These transcriptomic patterns associated with aging may be established in the brain early in adolescent [2]. Aging and aging-related diseases are thought to be centrally programmed by the hypothalamus [4,5]. Much evidence suggests that calorie restriction (CR), a non-invasive method to increase lifespan, has anti-inflammatory properties and reduces age-related oxidative stress [6–10]. A recent study suggests that CR leads to a neuroprotective transcriptomic profile in rodent brain and protects against aging related decline in memory function [11]. A large number of interacting genes, forming an interaction network, alter their transcription in response to aging and CR. However, understanding the complex interactions between genes is a challenge that cannot be addressed by looking at gene expression alone [12]. Network analyses of interactions between genes could further elaborate the complex mechanisms underlying both aging and CR. We can define interaction networks in several ways. Most often, we have represented gene interaction networks using correlations in gene expression. We can also derive from the same observations a measure of information shared between gene expressions [13] (reviewed in [14]). This flow of information between genes in a network therefore gives rise to new measurable characteristics (i.e. network behavior) which are absent in isolated components such as the expression levels of single genes (Alm 2003). Soltow *et al*. (2010) proposed that aging of a system can be generalized to four characteristics: (1) decline in capacity and utilization of energy, (2) decline in structure or metabolic organization, (3) decline in barrier functions and (4) decline in transfer of information [16]. The decline in structure and information flow during aging can be assessed by evaluating quantitative changes in network topology.

The structure of a gene network is subject to a few simple principles and allows for the comparison and characterization of different complex networks. Empirical studies show that many cellular networks are scale-free which means that the degree distribution (or connectivity of nodes) follows a negative power law [17]. In yeast exposed to CR, gene-regulatory networks were also found to be scale-free [18]. Many scale-free networks also have a hierarchical structure with high clustering coefficients of the nodes following a negative relationship with the degree distribution. These networks are found to divide naturally into highly connected clusters, which generally correspond to specific biological functions [19]. Biologically important genes play key topological roles in network function and structure, and influence network dynamic processes [20] enabling us to identify these central genes (network nodes) using network metrics such as node centrality. Central genes have different structural features such as having more connections, influencing more genes in the network through intermediary genes and playing a disproportionate role in information flow [21]. Eigenvector centrality captures the relative importance of each gene for the overall network structure, by reflecting the contribution of the variance in interaction of each gene to the overall interaction heterogeneity over the entire network [21].

Age-associated changes in network structure have been identified which can help us pinpoint the potential genome wide mechanisms involved in aging [16,22,23]. Network system failure is often associated with information flow disruptions and therefore central genes, in a gene interaction network, are expected to be particular foci for negative effects during the aging process [16]. Functionally-related genes should in principle co-express with each other and therefore gene co-expression networks, estimated using correlation in gene expression across individuals, should change with age. A key change in gene co-expression network structure associated with aging in mice was a loss in connectivity (26% decrease) over the entire network and a decrease in strength of correlations between genes within functional gene clusters [22]. Biologically-related genes clustered together forming highly connected functional gene clusters. Within these functional gene clusters there were gene groups involved in aging-related pathways [22]. Associations between interacting metabolites are also significantly altered under CR compared to *ad libitum* (AL) feeding in *Drosophila melanogaster* suggesting that network structure is modified under CR [23]. Laye *et al*. (2015) postulated that these changes in network structure under CR are protective against the age-associated decline in metabolic homeostasis. Aging protein-protein interaction networks of *D*. *melanogaster* showed that the relationship between identified protein clusters could be modified by CR [24]. These results suggest that aging-associated proteins might be key to maintaining stability in a network [24]. Southworth *et al*. (2009) identified the transcription factor nuclear factor-kappa B (NF-ĸB) as a central gene involved in the loss of network connectivity with aging in mice. Under CR, it would be anticipated that the network should be protected against the aging-associated decline in interdependencies, and should result in a less fragmented network [16].

Correlations between gene expression levels as a measure of gene interdependencies has been widely used to construct gene co-expression networks [25]. However, this method is limited in what can be inferred from these networks, as well as difficulties in identifying spurious correlations. Further, gene co-expression networks cannot separate direct interactions from transcriptional interactions that correlate with expression levels of many non-interacting genes. Therefore correlations cannot be used to reconstruct gene-gene interaction networks without additional and restrictive assumptions [26] and are heavily dependent on the associations being linear. In contrast, Mutual Information (MI) provides a measure of general dependence which is not necessarily assumed to be linear in form. MI provides an estimate of the information emanating from gene expression which is shared between any two given genes. This provides a mean to make inferences about interaction between genes in large networks [27,28]. The dependency found between two genes by MI-based methods represents a possible functional dependency, although causality cannot be inferred. Furthermore, MI is limited to only detect dependency between two genes. Conditional MI (CMI) estimates were developed, analogous to partial correlation coefficients for correlation measures, to discount the effects of the expression of other genes when estimating the MI for any gene pairs, hence accounting for false positives [29]. CMI is able to detect joint regulations of more than two genes by exploiting the conditional dependency between genes of interest. Therefore, CMI better captures functional relationships between genes and allows us to asses network-wide functional changes under, in our case, different CR levels [27,30,31]. Changes in a CMI network therefore represent changes in functions in response to aging. We can therefore capture overall functional changes concurrently, allowing us to detect central genes involved with these changes.

Based on the prior knowledge that aged brains have increased oxidative stress and inflammation [2,3], we tested whether the network structure of genes associated with aging, oxidative stress response and inflammation changed when individuals were exposed to different levels of CR. We postulated that the gene-gene CMI of these aging-associated genes would alter under CR, representing changes in functionality and regulation. We predicted that the network structure, specifically its connectivity would be increased by CR, which should protect against the loss of network connectivity. Furthermore, we expected that the changes in connectivity would be found in gene clusters, as demonstrated by Southworth et al. (2009), where aging-associated decline was typically found in connections between genes within functional gene clusters. We expected to see a change in central genes in the networks according to CR level, reflecting a potential protection against aging. Lastly, we also used classic Spearman's rank correlation coefficients to measure associations, and compared these results to those found using CMI. To test these predictions, we used the hypothalamic transcriptome of mice (20 weeks of age at the beginning of the experiment) exposed to three months graded CR (at levels up to 40% in 10% increments) [10,32–34] and constructed gene CMI interaction networks and gene co-expression networks of aging-associated genes for each CR level. This study complements previous work estimating the change in gene expression with CR for the hypothalamic transcriptome [34].

## RESULTS

### The effect of graded CR on the transcriptomic profile of aging-associated genes

**Figure 1:**
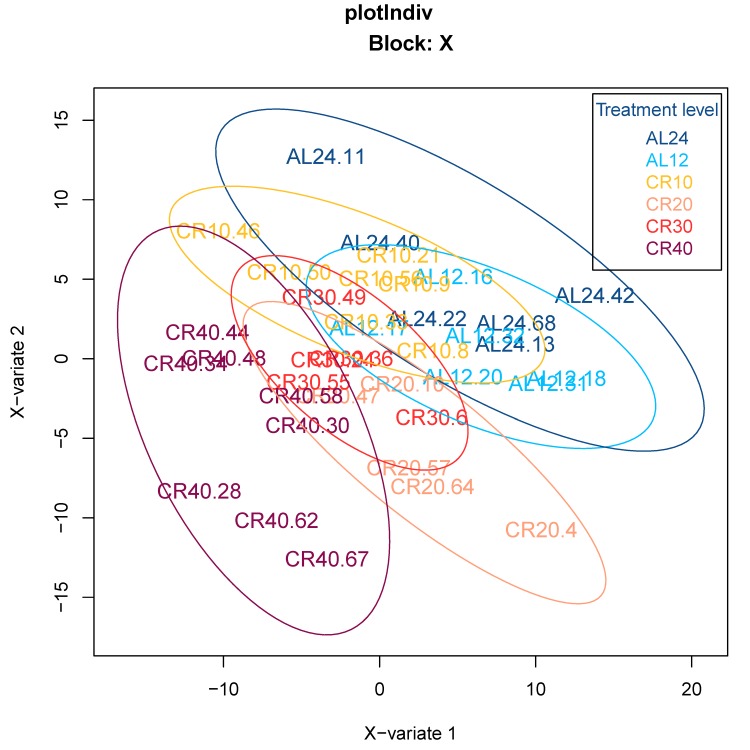
Orthogonal signal correction partial least squares discriminant analysis (O-PLS-DA) demonstrating the effect of graded CR on gene expression levels of aging-associated genes 24AL, 12AL, 10CR, 20CR, 30CR and 40CR refer respectively to 24h *ad libitum* feeding per day, 12h *ad libitum* feeding per day, 10 %, 20 %, 30 % and 40 % restriction.

We initially used an orthogonal signal correction partial least squares discriminant analysis (O-PLS-DA) to classify 12 and 24 hr AL fed groups (12AL, 24AL) and CR groups based on the expression levels of genes involved in aging, inflammation and oxidative stress (n = 408) obtained from *a priori* defined gene lists curated by Ingenuity Pathway Analysis (IPA) (‘inflammation of the nervous system’ and ‘oxidative stress response’) and GenAge database (‘aging-associated genes’ in *Mus musculus*). Individuals were clustered according to CR level, indicating that gene expression levels could predict CR level (Fig 1). Indeed model validation indicated that 41.8% of the variance in gene expression levels was explained by the different treatment groups (n = 6) (Xvar = 41.8 ± 18.5, p-value < 0.001). Of these 408 genes, expression analysis identified 16 genes differentially expressed at 24AL, 10 genes at 10CR, 14 genes at 20CR, 14 genes at 30CR and 59 genes at 40CR relative to 12AL (Table S1).

### The effect of graded CR on network structure and connectivity

**Figure 2:**
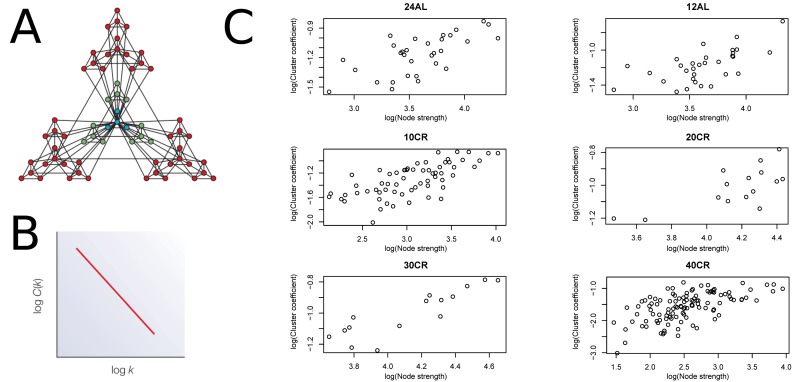
Node strength plotted against clustering coefficient to assess hierarchical topology (**A**) Visualization of a hierarchical network obtained from [12]. (**B**) Hierarchical network following a negative relationship between cluster coefficient and node strength. (**C**) Plots to assess hierarchical topology of aging-associated genes networks. 24AL, 12AL, 10CR, 20CR, 30CR and 40CR refer respectively to 24h *ad libitum* feeding per day, 12h *ad libitum* feeding per day, 10 %, 20 %, 30 % and 40 % restriction.

**Figure 3:**
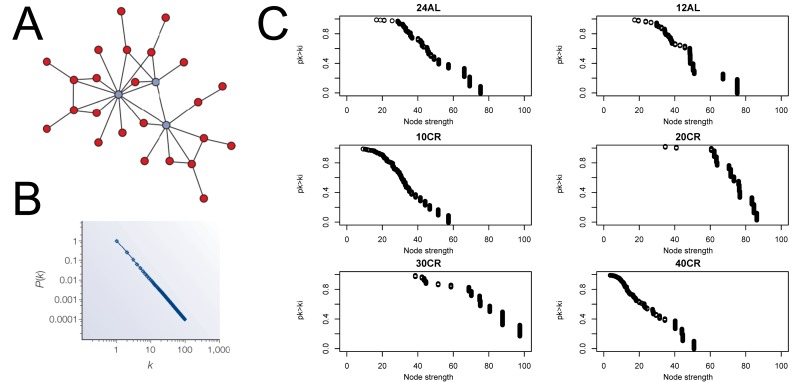
Node strength frequency distribution (**A**) Visualization of a scale free network obtained from [12]. (**B**) Node strength in scale free network follow a power law distribution [12]. (**C**) Plots to asses scale free topology of the aging-associated genes networks. 24AL, 12AL, 10CR, 20CR, 30CR and 40CR refer respectively to 24h *ad libitum feeding* per day, 12h *ad libitum* feeding per day, 10 %, 20 %, 30 % and 40 % restriction.

We estimated CMI based networks of these aging-associated genes and also standard gene co-expression networks based on correlations. CMI networks were characterized as having either a hierarchical or scale-free structure. Theory predicts that hierarchical networks should display a negative relationship between the degree to which genes cluster together (estimated as a cluster coefficient) and the number of neighbors of a given gene (node degree) (Fig 2A and 2B) [12]. The cluster coefficient did not decrease as node degree increased (Fig 2C), indicating that the networks were not hierarchical. Furthermore, node strength, a cumulative measure of node degree and the CMI of each gene, is predicted to follow a power distribution in scale-free networks (Fig 3A and 3B) [12]. However, in all networks node strength was not power law distributed (Fig 3C). In addition, visualizing the node strength on a log-log scale did not result in a linear relationship (Fig S1).

**Figure 4:**
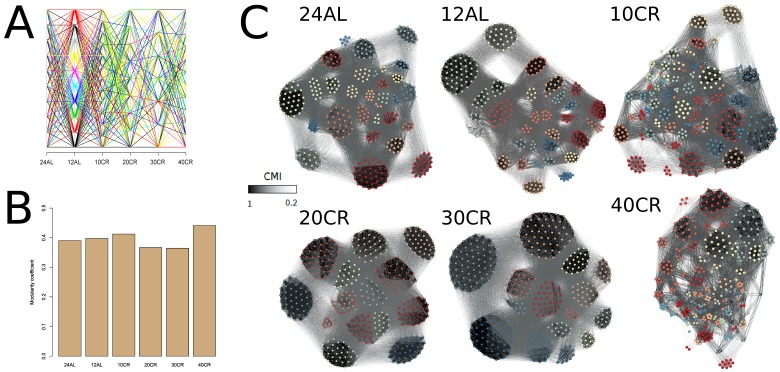
Modularity coefficients per conditional mutual information network at each treatment level were calculated and genes were assigned to clusters according to their mutual information (**A**) Changes of clusters across CR level and colored for clusters found at 12AL. (**B**) Modularity coefficient for each CMI network at each CR level. (**C**) CMI networks colored according to clusters identified at each CR level. Edges between genes with strong CMI are colored in black, those with weak CMI in light grey. 24AL, 12AL, 10CR, 20CR, 30CR and 40CR refer respectively to 24h *ad libitum feeding* per day, 12h *ad libitum* feeding per day, 10 %, 20 %, 30 % and 40 % restriction.

Another characterization of a network is the natural occurrence of gene clusters and the degree of connectivity of those clusters. This was estimated by modularity-based clusters of gene interactions and by assessing the strength of those interactions within these clusters. Modularity-based clusters of gene interactions changed and genes clustered differently at each CR level (Fig 4A) (Table S2). The modularity of the network, which represented the number of clusters, was also different at each CR level. The connectivity between genes in a cluster relative to the connections between clusters was measured by determining the modularity coefficient (Fig 4B). The highest modularity coefficient was observed at 10CR and 40CR, indicating that 10CR and 40CR had a higher connectivity between genes within clusters than between clusters (Fig 4C) (Table S2).

### The effects of graded CR on gene centrality

**Figure 5:**
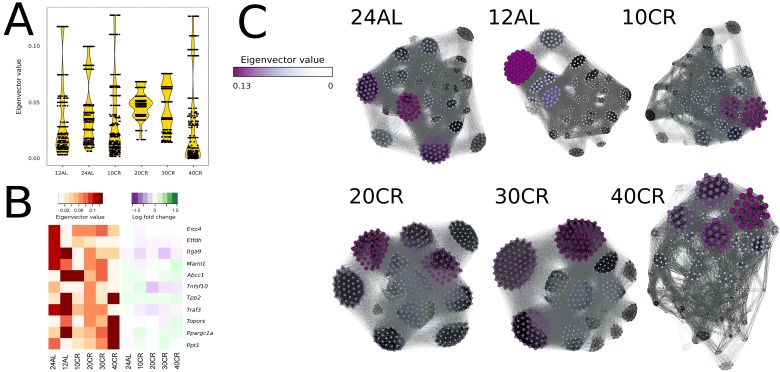
Node centrality was identified by calculating the eigenvector value for each gene in each conditional mutual information (CMI) network (**A**) Eigenvector distribution visualized in a violin plot per CR level. (**B**) Changes of eigenvector values and gene expression levels relative to 12h *ad libitum* feeding of key genes visualized in a heat map. (**C**) CMI networks at each CR level with node sizes and color proportional to eigenvector values. Edges between genes with strong CMI are colored in black, those with weak CMI in light grey. 24AL, 12AL, 10CR, 20CR, 30CR and 40CR refer respectively to 24h ad libitum feeding per day, 12h *ad libitum* feeding per day, 10 %, 20 %, 30 % and 40 % restriction.

We identified gene centrality, which are genes playing a central role in connectivity, by estimating gene eigenvector centrality in each CMI network. The variance in eigenvector centrality was reduced for 20CR and 30CR (Table S2) and we observed well-defined groups of genes with disproportionally large eigenvector centrality at 24AL, 12AL, 10CR and 40CR (Fig 5A) [35]. Central genes were identified based on the absolute maximum eigenvector centrality value (Table S2). At 24AL four genes were identified as central genes in the CMI network: excision repair cross-complementing rodent repair deficiency, complemen-tation group 4 (*Ercc4*), electron transferring flavoprotein, dehydrogenase (*Etfdh*), integrin alpha 9 (*Itga9*) and mastermind like 1 (Drosophila) (*Maml1*). One gene, ATP-binding cassette, sub-family C (CFTR/MRP), member 1 (*Abcc1*) was identified at 12AL, 10CR, and 30CR as a central gene. At 20CR three genes were identified as central genes: tumor necrosis factor (ligand) superfamily, member 10 (*Tnfsf10*), topoisomerase I binding, arginine/serine-rich (*Topors*), tripeptidyl peptidase II (*Tpp2*) and TNF receptor-associated factor 3 (*Traf3*). At 40CR the genes peroxisome proliferative activated receptor, gamma, coactivator 1 alpha (*Ppargc1a*) and palmitoyl-protein thioesterase 1 (*Ppt1*) were identified as central genes in the network. *Ppargc1a* and *Ppt1* showed a clear graded increase in centrality, and *Etfdh*, *Abbc1* and *Traf3* a de-crease in centrality relative to CR level, but all genes did not show any simultaneous significant changes in gene expression levels relative to 12AL (Fig 5B). Furthermore at all levels of CR, genes with the highest eigenvector values were found in the same clusters (Fig 5C).

### Comparison of networks based on CMI and correlation coefficients

We estimated Spearman's rank correlation coefficient between genes and constructed gene co-expression networks based on these correlations. The networks showed the same average number of edges between genes but their modularity and eigenvector centrality differed compared to CMI networks. Overall the gene co-expression network contained substantially more clusters compared to the CMI network with a higher modularity coefficient across CR. In contrast to the CMI based network, the modularity coefficient was the highest at 20CR (Table 1). The genes with the highest eigenvector centrality at 24AL, 20CR, 30CR and 40CR in the gene co-expression networks differed to CMI networks but were identical at 12AL and 10CR (*Abcc1*). Similar to the CMI networks, these genes were not significantly differentially expressed relative to 12AL. At 24AL angiotensinogen (serpin peptidase inhibitor, clade A, member 8) (*Agt*) was identified as central gene. In addition to *Abcc1*, four other genes were identified as central genes at 10CR: *Agt*, calcium/calmodulin-dependent protein kinase II gamma (*Camk2g*), CD200 antigen (*Cd200*), nuclear factor of activated T cells, cytoplasmic, calcineurin dependent 2 (*Nfatc2*) and transformation related protein 53 (*Trp53*). At 20 and 30CR the central gene was c-abl oncogene 1, non-receptor tyrosine kinase (*Abl1*). At 40CR the central gene was amyloid beta (A4) precursor protein (*App*).

**Table 1:** Comparison between gene co-expression networks and CMI networks

Treatment	Gene co-expression network		CMI network	
	Modularity coefficient	Clusters	Modularity coefficient	Clusters
24AL	0.751	21	0.390	10
12AL	0.729	21	0.397	10
10CR	0.749	31	0.412	13
20CR	0.774	13	0.367	9
30CR	0.732	14	0.364	7
40CR	0.727	21	0.441	12

Previous studies of gene expression networks in aging have mostly used gene co-expression networks to assess changes in network structure of individuals in relation to age and CR [16,22,23,36]. Here we used CMI because this measure provides a way to make inference about the amount of information shared between two genes when they are expressed. This is important for age-related studies for which we have a biological understanding that information flow at different cellular levels is disrupted by age. To our knowledge, CMI is used here for the first time to assess the changes in network topology associated with aging. We predicted that CR would result in a less fragmented regulatory network of aging associated genes compared to AL. We show that CR explained a significant proportion of the variability observed in the expression levels of aging-associated genes. Our CMI networks based on these genes suggest that (1) our networks were modular but did not have a hierarchical or scale-free topology; (2) the CMI network changed most at 40CR compared to 12AL with a higher connectivity between genes within clusters than between clusters; and (3) central genes for the regulatory network architecture showed a graded response to changing CR levels, with changes most pronounced at 40CR. This is consistent with previous studies at other biological levels for this experiment which showed a physiological state shift in mice exposed to 40CR [10,32–34,37]. Although the expression level of individual genes *Ppargc1a, Ppt1, Etfdh, Abbc1* and *Traf3* did not change [34], these genes were identified as ‘central genes’ indicating a network-wide role of these genes. We also showed that the networks exhibited a topological arrangement when exposed to graded CR.. It might be argued that when the networks are exposed to CR and close to a state shift, more non-monotonic relationships are formed which cannot be inferred using correlation methods.

## DISCUSSION

By comparing differences in network topology we can make inferences about changes in the functional interaction between genes that would not have been identified using a gene-by-gene traditional expression analysis.

### Regulatory networks of aging-associated genes exposed to graded CR have a modular topology

Many biological networks have a scale-free topology displaying a high degree of clustering which is suggested to be a consequence of a hierarchical organization of that network [17]. The CMI networks of our mice were not scale-free and did not display a hierarchical topology. Instead we had a modular network characterized by highly interlinked modules (or clusters) which were connected to each other with relatively few links [38]. Both the number of clusters and the gene composition of clusters changed in response to CR. These changes in gene cluster membership would suggest changes in functional interactions between genes in response to CR [18]. In agreement with our results, a study with *Caenorhabditis elegans* found a structural reorganization of the transcriptomic network when glucose metabolism was impaired, and specifically in aging-relevant signaling pathways including the mammalian target of rapamycin mTOR pathway [39]. We also found that as CR levels increased the number of connections between genes was higher within gene clusters compared to those between clusters. The changes in connectivity observed here indicate that at 10CR and 40CR the networks have a higher connectivity in these functional gene groups compared to the two AL groups. The differences observed in network behavior at 20CR and 30CR could be a representation of a phase transition resulting in a topological state shift occurring at 40CR [40]. This would be in agreement with phenomic state shifts observed for these mice [33,37,86]. Prolonged “network stress” was found to induce a topological phase transition in cellular networks with an entirely different switch in cellular function (reviewed in [41]). Exposing mice to CR imposes a “network stress” and structural reorganization is necessary as an adaptive response. The adaptive response to CR is well established to increase longevity in many taxa and induce other beneficial anti-aging effects (reviewed in [42]). A “network stress” induced shift includes an initial decrease in average link density which might help the network to prevent propagation of damage [43]. During prolonged “network stress”, the network undergoes a segregation between less important genes and more important genes (central genes) which results in these central genes displaying more and stronger links to their neighbors. This would lead to more highly connected clusters of genes and less connections between genes outside these clusters which was observed at 40CR. As the level of CR increased the level of “network stress” increased and segregation occurred resulting in a reorganization and higher connectivity within clusters at 40CR. The network reorganization and increased connectivity at 40CR suggests the network would be more protected against the aging-observed decline in connectivity [22].

### Changes in gene centrality irrespective of gene expression levels

Topologically central genes have a disproportionate influence on the overall integrity of gene regulatory networks [20]. Here, we identified central genes that played a central role in network structure and found a change in the connectivity of these genes in relation to the CR level. The central gene in the CMI networks at 12AL, 10CR and 30CR was *Abcc1* or also known as MRP1. The importance of this gene in the network structure deceased in a graded manner with increasing CR level. Gene expression analysis of this gene showed no significant difference of this gene under CR compared to 12AL. This is in agreement with Laye *et al*. (2015) who demonstrated that the changes in correlation coefficient with age between metabolites in a co-expression network were independent of their mean levels [23]. Although first identified as a drug transporter, MRP1 is also involved in inflammation and oxidative stress [44]. MRP1 can also flux small peptides such as N-acetyl-leu-leu-norleucina (ANLL) which inhibits the 6S-proteasome activity which activates NF-ĸB [45]. Furthermore TNF-α has been associated with MRP1 [46]. We previously found that circulating TNF-α levels were reduced under CR [10] and that the transcription factor NF-ĸB in the hypothalamus was inhibited at all CR levels compared to AL, suggesting a reduced state of inflammation [34]. Furthermore micro-inflammation in the hypothalamus involving elevated NF-ĸB was recently suggested to mediate whole body-aging [5] and therefore a reduced state of inflammation under CR would have a protective role against whole-body aging.

At 20CR four genes were identified as central genes in network structure: *Tnfsf10*, *Traf3*, *Tpp2* and *Topors*. The genes *Tnfsf10* and *Traf3* are associated with TNF-α and we established previously that *Tnfsf10* correlated positively with circulating TNF-α levels [10,34]. Interestingly in human centenarians dementia is associated with high levels of circulating TNF-α levels and suggests a role of TNF-α in age-associated brain pathology [47]. Our results may indicate that reduced TNF-α signaling via *Tnfsf10* and *Traf3* plays a key role in network structure at 20CR. The other key gene, *Tpp2*, was shown to have a pro-longevity effect based on a mouse knockout study [48]. *Tpp2* knockout mice over 1 year old exhibited elevated mortality associated with an aged appearance. Furthermore, alterations were observed in p53 expression and NF-ĸB activation but no direct molecular link was found with *Tpp2* [48]. *Tnfsf10* has also been found to mediate p53 dependent cell death [49]. Similar to *Tpp2*, *Topors* was also identified as having a pro-longevity effect [50] and also found to act as a tumor suppressor [51]. *Topors* knockout mice exhibited a significant reduction in mean and maximum lifespan and several mice had signs of premature aging [50].

The connectivity of the 40CR network differed substantially from the 12AL network. If our assertions are right, the 40CR network represents a network which receives the benefits of CR. *Ppargc1a* and *Ppt1* were key topological central genes at 40CR which were involved in this overall difference in connectivity. They are important to protect the network from aging-associated loss in structure and fragmentation. A key change associated with aging was a loss in connectivity over the entire network and a decrease between genes within functional gene clusters [22]. Central genes are therefore expected to be particular foci for negative effects during the aging process [16]. *Ppargc1a* encodes PGC1α which is a major transcription factor contributing to gluconeogenesis, energy metabolism, lipid metabolism and functioning of the mitochondria [52]. PGC1α was shown to be upregulated in the brain when exposed to long term CR [53] but not in the hypothalamus when fasted for 60h [54]. Here, we did not find a significant regulation of *Ppargc1a* under CR compared to 12AL after 3 months of study. Brain aging was associated with a downregulation of the PGC1α mediated transcriptional pathway [55] and neuro-degenerative disorders such as Parkinson's disease are associated with lower levels of the target genes of PGC1α [56]. In Alzheimer's patients PGC1α expression is reduced [57]. Interestingly PGC1α overexpression in transgenic mice models for Alzheimer diseases exacerbates the neuropathological and behavioral deficits [58]. PGC1α null mice are lean and are resistant to diet-induced obesity. These mice exhibit profound hyperactivity associated with lesions in the brain region that controls movement [59]. When PGC1α was blocked locally within the central nervous system, fasted rats exhibited lower 24h food intake compared to those treated with a vehicle. Similar results were found for non-fasted high-fat induced obese rats but not for non-fasted low fat chow fed rats suggesting a greater role for neural PGC1α during fasting and obesity [60]. Fasted neural PGC1α null mice show diminished hypothalamic expression levels of the neuropeptides agouti-related neuropeptide (AgRP) and neuropeptide Y (NPY) which would suggest a role of PGC1α in regulating energy metabolism via regulation of hypothalamic neuropeptides [61]. The neuropeptides AgRP and NPY both increase hunger signaling and were upregulated in our transcriptomic dataset [34]. Although shown in diet induced obese mice, PGC1α is also associated with the control of reactive oxygen species (ROS) by hypothalamic pro-opiomelanocortin (POMC) neurons and hypothalamic ROS levels are positively correlated with circulating leptin levels [62]. Diano et al. (2011) postulated that the leptin resistance observed in these mice might be controlled by ROS manifested by reduced POMC and increased NPY/AgRP neuronal firing [62]. Interestingly during CR, leptin levels are reduced and correlated positively with reduced expression levels of POMC and negatively with increased expression levels of NPY/AgRP in our dataset [10,34] which suggests an improved leptin sensitivity and reduced levels of ROS. However, because of a lack of tissue we were unable to measure hypothalamic ROS levels in this study but we can conclude that our mice exhibited an increased ‘hunger profile’ [34]. Upon re-feeding, after a period of short-term (100 days) CR, the hyperphagic response suggests that hunger remained even after energy balance was re-established [63]. This elevated hunger profile might be a major factor contributing to the beneficial effect of CR [64]. A recent study has shown an abrogated effect on longevity when NPY^−/−^ mice were exposed to 30% CR compared to the AL fed mice, key role for NPY to link CR to increased lifespan [65].

We and others have previously postulated that hypothalamic nutrient sensing signaling might mediate the observed increase in lifespan under CR treatment [34,66–68]. Mice lacking PGC1α have abnormal diurnal rhythms of activity and metabolic rate and this was related to abnormalities in expression of clock genes and energy metabolism associate genes [69]. PGC1α also induces circadian clock resetting in restricted feeding and stimulates the clock gene aryl hydrocarbon receptor nuclear translocator-like (ARNTL) expression [69]. transcriptomic analysis and others indeed suggests that under CR mice are protected against aging-associated desynchronization of the circadian clock [34,36,70,71]. Although *Ppargc1a* gene expression levels were not significantly correlated with the extent of restriction, its downstream effects were. Here we were able to elucidate the importance of this gene in metabolic regulation via network analysis which would have remained unidentified with simple gene by gene expression based approaches. Interestingly mice with *Ppt1* deficiency, the other key gene that changed in relation to the degree of CR, exhibited disruption of adaptive energy metabolism and downregulation of PGC1α [72]. Due to their key roles in network structure as central genes, *Ppargc1a* and *Ppt1* play a major role in protecting the network from age-observed decline in connectivity [22,23].

### Comparison of CMI and correlation coefficient based networks

Gene co-expression networks based on correlation coefficients have been widely used, for example in aging/CR research [16,23,36,73], to assess topological changes to networks in response to diseases [74–79], as characterization of a cellular state [80] and to understand plant cellular processes [81,82]. In contrast, CMI based networks have rarely been used despite recent theoretical advances in their application and interpretation and additional insight they can provide. Correlations assess ‘co-expression’ as the sum of the product of two random variables, while CMI specifically calculates the sum of the joint probabilities of two random variables from which inferences of causality can be made. Crucially, correlation assumes linear associations, or associations that change at the same rate with rank/order for non-parametric measures. CMI however, will detect associations whether they are monotonic or not. These measures are not antagonistic, they are complementary [83]. However, a major hurdle arises when we try to address the issue that in a many-body system the association between any given pair of objects can be influenced by their own associations with other objects. In our case this represents our attempt to detect whether the co-expression of two genes is real or caused by their mutual co-expression with a third gene. CMI estimates are more robust than partial correlation estimates because we have a better understanding of how to define joint probability distributions. Therefore, CMI can be more advantageous, particularly for large networks [84]. Finally correlation and CMI capture the shared information/covariance between two different measures of gene expression. For correlation, we try to understand the shared variance in gene expression. With CMI we try to estimate the joint information emanating from genes and thereby assuming causality. These two methods have previously been compared and an almost one-to-one correspondence between the two methods was found, the authors concluding that non-linear relationships did not play an important role in their gene networks [83,85]. The fundamental difference however lies in what can be inferred from these two methods. We indeed found similarities between our CMI and gene co-expression networks with respect to eigenvector centrality at 12AL and 10CR but we also found fundamental differences in modularity. In the CMI network the intermediate groups had fewer clusters but the amount of clusters at 40CR was more similar to 12AL while modularity differed. This was clearly shown by the modularity coefficient of the CMI based analysis while this was not the case for the correlation coefficient based networks. We can conclude from these results that gene co-expression based modularity coefficient estimates were relatively insensitive to CR whereas the CMI estimates were able to discriminate between 20CR and 30CR. It might be argued that when the networks are exposed to CR and close to a state shift, more non-monotonic relationships are formed which cannot be inferred using correlation methods. Hence CMI provides a measure for which we can directly test biologically relevant co-expression and given the particular focus we can measure changes in information flow and regulation with aging and CR.

In this study we found a structural reorganization of the gene regulatory network under graded levels of CR. Using the CMI network analysis approach we identified *Ppargc1a, Ppargc1a*, *Ppt1*, *Etfdh*, *Traf3* and *Abcc1* as key regulatory genes in network structure with changes in their centrality as the level of CR increased. Since expression levels of these genes did not change with CR, this study emphasizes the importance to move beyond gene by gene level analyses to better understand regulatory changes of the transcriptome when it is challenged (in our case by CR). Our results suggest that the network structure of aging-associated genes under graded CR was altered and might play an important role in preventing the loss of network structure observed with age.

## MATERIALS AND METHODS

To create a systems level description of graded CR responses, we performed a three month graded CR study on male C57BL/6 mice commencing at age 5 months [10,32,33,37,86]. Full details of the experimental protocol and rationale for the experiment are given in [32] and are elaborated briefly below. In this study, transcriptome sequencing or RNA-seq of the hypothalamus (see also [34]) was used to assess changes in network structure of aging-associated genes.

### Animals and experimental manipulations

All procedures were approved by the University of Aberdeen ethical approval committee and carried out under the Animals (Scientific Procedures) Act 1986 Home Office license (PPL 60/4366 held by JRS). Forty nine male C57BL/6 mice (*Mus musculus*), purchased from Charles River (Ormiston, UK) were individually housed and free access to water was provided. Mice were exposed to 12 hour dark/light cycle (lights on at 0630h) and body mass and food intake were recorded daily, immediately prior to nocturnal feeding. At 20 weeks of age (resembling early adulthood in humans), mice had their food intake monitored for 2 weeks of baseline and were then randomly allocated into 6 different treatment groups: 24h *ad libitum* intake (24AL) (n=8), 12AL intake which had access to *ad libitum* food but for only for the 12 hours of darkness per day, sometimes also called ‘time restricted feeding’ (n=8), 10 CR (n=8), 20CR (n=8), 30CR (n=8) and 40CR (n=9). Mice exposed to 40CR indicates 40% fewer calories were provided than the individual intake measured over a baseline period of 14 days prior to introducing CR. This is a CR protocol rather than a caloric dilution experiment [87].

Animals fed completely AL (i.e., having 24 hours access to food) may potentially over feed, become obese and CR associated changes compared to 24AL would therefore most likely reflect the anti-obesity effect of CR [42,88]. To address this issue, 12AL was set as a reference and graded levels of CR were introduced to investigate a potential graded response mirroring the graded lifespan response to CR manipulation [87,89]. Detailed information on overall study design, diet composition and detailed rationale are described elsewhere [32].

### RNA isolation, RNA-sequencing, alignment and analytical procedure

After culling by a terminal CO_2_ overdose, brains were removed, weighed and frozen in isopentane over dry ice and stored at −80°C for RNA isolation. The hypothalamus was carefully dissected at a later stage and RNA was isolated by homogenizing in Trizol (Sigma Aldrich, UK) according to manufacturer's instructions. Prior to RNA quantification, using the Agilent RNA 6000 Nano Kit, samples were denatured at 65°C.

Due to the very small size of the hypothalamus, some samples did not contain sufficient quantity of high quality RNA. In total, the RNA of 37 individual mice (12 h AL n=6, 24 h AL n=6, 10 % CR n=7, 20 % CR n=5, 30 % CR n=5, 40 % CR n=8) was successfully isolated and sent to Beijing Genomic Institute (BGI, Hong Kong) for RNA sequencing. Library preparation was done using a standard protocol of BGI and the library products were sequenced using an Illumina Hi-seq 2000, resulting in 50 bp single end reads. Standard primers and barcodes developed by BGI were used. Detailed information on library preparation for these samples and the bio-informatic pipeline has been described previously [34].

### Network construction and biological interpretation

Based on the prior knowledge that aged brains have increased oxidative stress and inflammation [2,3], we curated a data set based on *a prior* defined gene lists from Ingenuity pathway analysis (IPA) (Ingenuity® Systems, http://www.ingenuity.com/products/ipa,version 2000-2015): ‘inflammation of the nervous system’ (n=196) and ‘oxidative stress response’ (n=155); and genes associated with aging derived from the online database GenAge (build 18, version 11/10/2015) (n=99) [48]. Normalized read counts (for method see [34]) of these *a priori* identified gene were initially analyzed by an O-PLS-DA. The Devium package remove the first package with PLS and OPLS R command functions were used (https://github.com/dgrapov/devium.git) to validate the model in the statistical environment R (version 3.1.2) [90].

The Bioconductor package minet [91] was used to infer large transcriptomic networks using CMI. The package returns a network where the nodes are genes and the edges are statistical dependencies between genes (CMI matrix). Four different entropy estimators and four different inference methods are available of which we used the Miller-Madow asymptotic bias corrected empirical estimator [92] and the ARACNE method [26]. CMI between genes was inferred in two steps: (1) MI is estimated between all pairs of genes and (2) the ARACNE inference algorithm applies a filter to remove estimated spurious links. ARACNE initially assigns to each pair of genes (i.e. nodes) a weight equal to MI and then removes any indirect interaction [26]. A network based on Spearman correlation coefficients between gene expression levels were also estimated with minet.

Gene centrality measures were estimated for each network based on eigenvector values. Eigenvector centrality captures the relative importance of each gene for the overall interaction network structure by estimating the contribution of the variance in interaction of each gene to the overall interaction heterogeneity in the entire network [21]. Gene eigenvector centrality was estimated by Eigen-decomposing each CMI network and using the elements of the eigenvector corresponding to the dominant eigenvalue from these decompositions. Gene strength per gene was calculated by the sum of the CMI between that gene and other genes in the network (row sums of the CMI matrix).

Weighted clustering coefficient estimates were used as described in [93]. Clusters of genes were identified based on defining a parsimonious division of a network [94,95]. This approach maximizes the number of connections (edges) within a cluster and minimizes the number of connections between clusters. The modularity coefficient quantifies the modularity technique and is a sum of associations for all genes belonging to the same clusters minus its expected value if genes were associated at random, based on the strength of each gene (method based on [93]).

## SUPPLEMENTARTY DATA



## References

[1] Yankner BA (2000). A century of cognitive decline. Nature.

[2] Lu T, Pan Y, Kao S-Y, Li C, Kohane I, Chan J, Yankner BA (2004). Gene regulation and DNA damage in the ageing human brain. Nature.

[3] Lee CK, Weindruch R, Prolla TA (2000). Gene-expression profile of the ageing brain in mice. Nat Genet.

[4] Gabuzda D, Yankner B (2013). Physiology: Inflammation links ageing to the brain. Nature.

[5] Zhang G, Li J, Purkayastha S, Tang Y, Zhang H, Yin Y, Li B, Liu G, Cai D (2013). Hypothalamic programming of systemic ageing involving IKK-β, NF-κB and GnRH. Nature.

[6] Hyun D-H, Emerson SS, Jo D-G, Mattson MP, de Cabo R (2006). Calorie restriction up-regulates the plasma membrane redox system in brain cells and suppresses oxidative stress during aging. Proc Natl Acad Sci U S A.

[7] Chung HY, Kim HJ, Kim KW, Choi JS, Yu BP (2002). Molecular inflammation hypothesis of aging based on the anti-aging mechanism of calorie restriction. Microsc Res Tech.

[8] Sohal RS, Agarwal S, Candas M, Forster MJ, Lal H (1994). Effect of age and caloric restriction on DNA oxidative damage in different tissues of C57BL/6 mice. Mech Ageing Dev.

[9] López-Torres M, Barja G (2008). Calorie restriction, oxidative stress and longevity. Rev Esp Geriatr Gerontol.

[10] Mitchell SE, Delville C, Konstantopedos P, Hurst J, Derous D, Green C, Chen L, Han JJD, Wang Y, Promislow DEL, Lusseau D, Douglas A, Speakman JR (2015). The effects of graded levels of calorie restriction: II. Impact of short term calorie and protein restriction on circulating hormone levels, glucose homeostasis and oxidative stress in male C57BL/6 mice. Oncotarget.

[11] Wood SH, van Dam S, Craig T, Tacutu R, O'Toole A, Merry BJ, de Magalhães JP (2015). Transcriptome analysis in calorie-restricted rats implicates epigenetic and post-translational mechanisms in neuroprotection and aging. Genome Biol.

[12] Barabási A-L, Oltvai ZN (2004). Network biology: understanding the cell's functional organization. Nat Rev Genet.

[13] Shannon CE (1948). A mathematical theory of communication. Bell Syst Tech J.

[14] Tkačik G, Walczak AM (2011). Information transmission in genetic regulatory networks: a review. J Phys Condens Matter.

[15] Alm E, Arkin AP (2003). Biological networks. Curr Opin Struct Biol.

[16] Soltow QA, Jones DP, Promislow DEL (2010). A network perspective on metabolism and aging. Integr Comp Biol.

[17] Arenas A, Cabrales A, Diaz-Guilera A, Guimera R, Vega-Redondo F, Pastor-Satorras R, Rubi M, Diaz-Guilera A (2003). Search and Congestion in Complex Networks. Stat Mech Complex Networks Lect Notes Phys.

[18] Wuttke D, Connor R, Vora C, Craig T, Li Y, Wood S, Vasieva O, Shmookler Reis R, Tang F, de Magalhães JP, Kim SK (2012). Dissecting the Gene Network of Dietary Restriction to Identify Evolutionarily Conserved Pathways and New Functional Genes. PLoS Genet.

[19] Eisen MB, Spellman PT, Brown PO, Botstein D (1998). Cluster analysis and display of genome-wide expression patterns. Proc Natl Acad Sci.

[20] Hu P, Fan W, Mei S (2015). Identifying node importance in complex networks. Phys A Stat Mech its Appl.

[21] Newman M (2010). Networks: An Introduction.

[22] Southworth LK, Owen AB, Kim SK (2009). Aging mice show a decreasing correlation of gene expression within genetic modules. PLoS Genet.

[23] Laye MJ, Tran V, Jones DP, Kapahi P, Promislow DEL (2015). The effects of age and dietary restriction on the tissue-specific metabolome of Drosophila. Aging Cell.

[24] Xue H, Xian B, Dong D, Xia K, Zhu S, Zhang Z, Hou L, Zhang Q, Zhang Y (2007). A modular network model of aging. Mol Syst Biol.

[25] Zhao W, Langfelder P, Fuller T, Dong J, Li A, Hovarth S (2010). Weighted gene coexpression network analysis: state of the art. J Biopharm Stat.

[26] Margolin AA, Nemenman I, Basso K, Wiggins C, Stolovitzky G, Dalla Favera R, Califano A (2006). ARACNE: an algorithm for the reconstruction of gene regulatory networks in a mammalian cellular context. BMC Bioinformatics.

[27] Zhang X, Zhao X-M, He K, Lu L, Cao Y, Liu J, Hao J-K, Liu Z-P, Chen L (2012). Inferring Gene Regulatory Networks from Gene Expression Data by Path Consistency Algorithm Based on Conditional Mutual Information. Bioinformatics.

[28] Kinney JB, Atwal GS (2014). Equitability, mutual information, and the maximal information coefficient. Proc Natl Acad Sci U S A.

[29] Frenzel S, Pompe B (2007). Partial Mutual Information for Coupling Analysis of Multivariate Time Series. Phys Rev Lett.

[30] Liang K-C, Wang X (2008). Gene regulatory network reconstruction using conditional mutual information. EURASIP J Bioinform Syst Biol.

[31] Li X, Ouyang G (2010). Estimating coupling direction between neuronal populations with permutation conditional mutual information. Neuroimage.

[32] Mitchell SE, Tang Z, Kerbois C, Delville C, Konstantopedos P, Bruel A, Derous D, Green C, Aspden RM, Goodyear SR, Chen L, Han JJD, Wang Y (2015). The effects of graded levels of calorie restriction: I. impact of short term calorie and protein restriction on body composition in the C57BL/6 mouse. Oncotarget.

[33] Mitchell SE, Delville C, Konstantopedos P, Derous D, Green CL, Chen L, Han J-DJ, Wang Y, Promislow DEL, Douglas A, Lusseau D, Speakman JR (2015). The effects of graded levels of calorie restriction: III. Impact of short term calorie and protein restriction on mean daily body temperature and torpor use in the C57BL/6 mouse. Oncotarget.

[34] Derous D, Mitchell SE, Green CL, Chen L, Han J-DJ, Wang Y, Promislow DEL, Lusseau D, Speakman JR, Douglas A (2016). The effects of graded levels of calorie restriction: VI. Impact of short-term graded calorie restriction on transcriptomic responses of the hypothalamic hunger and circadian signaling pathways. Aging (Albany NY).

[35] Martin T, Zhang X, Newman MEJ (2014). Localization and centrality in networks. Phys Rev E.

[36] Plank M, Wuttke D, van Dam S, Clarke SA, de Magalhães JP (2012). A meta-analysis of caloric restriction gene expression profiles to infer common signatures and regulatory mechanisms. Mol Biosyst.

[37] Lusseau D, Mitchell SE, Barros C, Derous D, Green C, Chen L, Han J-DJ, Wang Y, Promislow DEL, Douglas A, Speakman JR (2015). The effects of graded levels of calorie restriction: IV. Non-linear change in behavioural phenotype of mice in response to short-term calorie restriction. Sci Rep.

[38] Ravasz E, Somera AL, Mongru DA, Oltvai ZN, Barabási AL (2002). Hierarchical organization of modularity in metabolic networks. Science.

[39] Priebe S, Menzel U, Zarse K, Groth M, Platzer M, Ristow M, Guthke R (2013). Extension of life span by impaired glucose metabolism in Caenorhabditis elegans is accompanied by structural rearrangements of the transcriptomic network. PLoS One.

[40] Chen L, Liu R, Liu Z-P, Li M, Aihara K (2012). Detecting early-warning signals for sudden deterioration of complex diseases by dynamical network biomarkers. Sci Rep.

[41] Szalay MS, Kovács IA, Korcsmáros T, Böde C, Csermely P (2007). Stress-induced rearrangements of cellular networks: Consequences for protection and drug design. FEBS Lett.

[42] Speakman JR, Mitchell SE (2011). Caloric restriction. Molecular Aspects of Medicine.

[43] Motter AE (2004). Cascade control and defense in complex networks. Phys Rev Lett.

[44] Cole SPC (2014). Multidrug resistance protein 1 (MRP1, ABCC1), a “multitasking” ATP-binding cassette (ABC) transporter. J Biol Chem.

[45] Jansen G, Scheper R, Dijkmans B (2003). Multidrug resistance proteins in rheumatoid arthritis, role in disease-modifying antirheumatic drug efficacy and inflammatory processes: an overview. Scand J Rheumatol.

[46] Stein U, Walther W, Laurencot CM, Scheffer GL, Scheper RJ, Shoemaker RH (1997). Tumor necrosis factor-alpha and expression of the multidrug resistance-associated genes LRP and MRP. J Natl Cancer Inst.

[47] Bruunsgaard H, Andersen-Ranberg K, Jeune B, Pedersen AN, Skinhøj P, Pedersen BK (1999). A high plasma concentration of TNF-alpha is associated with dementia in centenarians. J Gerontol A Biol Sci Med Sci.

[48] Tacutu R, Craig T, Budovsky A, Wuttke D, Lehmann G, Taranukha D, Costa J, Fraifeld VE, de Magalhães JP (2013). Human Ageing Genomic Resources: integrated databases and tools for the biology and genetics of ageing. Nucleic Acids Res.

[49] Kuribayashi K, Krigsfeld G, Wang W, Xu J, Mayes PA, Dicker DT, Wu GS, El-Deiry WS (2008). TNFSF10 (TRAIL), a p53 target gene that mediates p53-dependent cell death. Cancer Biol Ther.

[50] Marshall H, Bhaumik M, Aviv H, Moore D, Yao M, Dutta J, Rahim H, Gounder M, Ganesan S, Saleem A, Rubin E (2010). Deficiency of the dual ubiquitin/SUMO ligase Topors results in genetic instability and an increased rate of malignancy in mice. BMC Mol Biol.

[51] Saleem A, Dutta J, Malegaonkar D, Rasheed F, Rasheed Z, Rajendra R, Marshall H, Luo M, Li H, Rubin EH (2004). The topoisomerase I- and p53-binding protein topors is differentially expressed in normal and malignant human tissues and may function as a tumor suppressor. Oncogene.

[52] Kersten S, Desvergne B, Wahli W (2000). Roles of PPARs in health and disease. Nature.

[53] Ranhotra HS (2010). Long-term caloric restriction up-regulates PPAR gamma co-activator 1 alpha (PGC-1alpha) expression in mice. Indian J Biochem Biophys.

[54] Tritos N a, Mastaitis JW, Kokkotou EG, Puigserver P, Spiegelman BM, Maratos-Flier E (2003). Characterization of the peroxisome proliferator activated receptor coactivator 1 alpha (PGC 1alpha) expression in the murine brain. Brain Res.

[55] Jiang T, Yin F, Yao J, Brinton RD, Cadenas E (2013). Lipoic acid restores age-associated impairment of brain energy metabolism through the modulation of Akt/JNK signaling and PGC1α transcriptional pathway. Aging Cell.

[56] Zheng B, Liao Z, Locascio JJ, Lesniak KA, Roderick SS, Watt ML, Eklund AC, Zhang-James Y, Kim PD, Hauser MA, Grünblatt E, Moran LB, Mandel SA (2010). PGC-1α, a potential therapeutic target for early intervention in Parkinson's disease. Sci Transl Med.

[57] Qin W, Haroutunian V, Katsel P, Cardozo CP, Ho L, Buxbaum JD, Pasinetti GM (2009). PGC-1alpha expression decreases in the Alzheimer disease brain as a function of dementia. Arch Neurol.

[58] Dumont M, Stack C, Elipenahli C, Jainuddin S, Launay N, Gerges M, Starkova N, Starkov A a, Calingasan NY, Tampellini D, Pujol A, Beal MF (2014). PGC-1α overexpression exacerbates β-amyloid and tau deposition in a transgenic mouse model of Alzheimer's disease. FASEB J.

[59] Lin J, Wu PH, Tarr PT, Lindenberg KS, St-Pierre J, Zhang CY, Mootha VK, Jäger S, Vianna CR, Reznick RM, Cui L, Manieri M, Donovan MX (2004). Defects in adaptive energy metabolism with CNS-linked hyperactivity in PGC-1α null mice. Cell.

[60] Ryan KK, Li B, Grayson BE, Matter EK, Woods SC, Seeley RJ (2011). A role for central nervous system PPAR-γ in the regulation of energy balance. Nat Med.

[61] Ma D, Li S, Lucas EK, Cowell RM, Lin JD (2010). Neuronal inactivation of peroxisome proliferator-activated receptor γ coactivator 1α (PGC-1α) protects mice from diet-induced obesity and leads to degenerative lesions. J Biol Chem.

[62] Diano S, Liu Z-W, Jeong JK, Dietrich MO, Ruan H-B, Kim E, Suyama S, Kelly K, Gyengesi E, Arbiser JL, Belsham DD, Sarruf D a, Schwartz MW (2011). Peroxisome proliferation-associated control of reactive oxygen species sets melanocortin tone and feeding in diet-induced obesity. Nat Med.

[63] Hambly C, Mercer JG, Speakman JR (2007). Hunger does not diminish over time in mice under protracted caloric restriction. Rejuvenation Res.

[64] Chiba T, Yamaza H, Higami Y, Shimokawa I (2002). Anti-aging effects of caloric restriction: Involvement of neuroendocrine adaptation by peripheral signaling. Microsc Res Tech.

[65] Chiba T, Tamashiro Y, Park D, Kusudo T, Fujie R, Komatsu T, Kim SE, Park S, Hayashi H, Mori R, Yamashita H, Chung HY, Shimokawa I (2014). A key role for neuropeptide Y in lifespan extension and cancer suppression via dietary restriction. Sci Rep.

[66] Libert S, Zwiener J, Chu X, Vanvoorhies W, Roman G, Pletcher SD (2007). Regulation of Drosophila life span by olfaction and food-derived odors. Science.

[67] Bishop NA, Guarente L (2007). Two neurons mediate diet-restriction-induced longevity in C. elegans. Nature.

[68] Broughton SJ, Piper MDW, Ikeya T, Bass TM, Jacobson J, Driege Y, Martinez P, Hafen E, Withers DJ, Leevers SJ, Partridge L (2005). Longer lifespan, altered metabolism, and stress resistance in Drosophila from ablation of cells making insulin-like ligands. Proc Natl Acad Sci U S A.

[69] Liu C, Li S, Liu T, Borjigin J, Lin JD (2007). Transcriptional coactivator PGC-1alpha integrates the mammalian clock and energy metabolism. Nature.

[70] Wyse CA, Coogan AN, Selman C, Hazlerigg DG, Speakman JR (2010). Association between mammalian lifespan and circadian free-running period: the circadian resonance hypothesis revisited. Biol Lett.

[71] Swindell WR (2008). Comparative analysis of microarray data identifies common responses to caloric restriction among mouse tissues. Mech Ageing Dev.

[72] Wei H, Zhang Z, Saha A, Peng S, Chandra G, Quezado Z, Mukherjee AB (2011). Disruption of adaptive energy metabolism and elevated ribosomal p-S6K1 levels contribute to INCL pathogenesis: Partial rescue by resveratrol. Hum Mol Genet.

[73] van Dam S, Cordeiro R, Craig T, van Dam J, Wood SH, de Magalhães J (2012). GeneFriends: An online co-expression analysis tool to identify novel gene targets for aging and complex diseases. BMC Genomics.

[74] Presson AP, Sobel EM, Papp JC, Suarez CJ, Whistler T, Rajeevan MS, Vernon SD, Horvath S (2008). Integrated Weighted Gene Co-expression Network Analysis with an Application to Chronic Fatigue Syndrome. BMC Syst Biol.

[75] Dobrin R, Zhu J, Molony C, Argman C, Parrish ML, Carlson S, Allan MF, Pomp D, Schadt EE (2009). Multi-tissue coexpression networks reveal unexpected subnetworks associated with disease. Genome Biol.

[76] Saris CGJ, Horvath S, van Vught PWJ, van Es M a, Blauw HM, Fuller TF, Langfelder P, DeYoung J, Wokke JHJ, Veldink JH, van den Berg LH, Ophoff R a (2009). Weighted gene co-expression network analysis of the peripheral blood from Amyotrophic Lateral Sclerosis patients. BMC Genomics.

[77] Zhang J, Xiang Y, Ding L, Keen-Circle K, Borlawsky TB, Ozer HG, Jin R, Payne P, Huang K (2010). Using gene co-expression network analysis to predict biomarkers for chronic lymphocytic leukemia. BMC Bioinformatics.

[78] Ray M, Zhang W (2010). Analysis of Alzheimer's disease severity across brain regions by topological analysis of gene co-expression networks. BMC Syst Biol.

[79] Bettencourt C, Forabosco P, Wiethoff S, Heidari M, Johnstone DM, Botía JA, Collingwood JF, Hardy J, Milward EA, Ryten M, Houlden H (2016). Gene co-expression networks shed light into diseases of brain iron accumulation. Neurobiol Dis.

[80] Carter SL, Brechbuhler CM, Griffin M, Bond AT (2004). Gene co-expression network topology provides a framework for molecular characterization of cellular state. Bioinformatics.

[81] Aoki K, Ogata Y, Shibata D (2007). Approaches for extracting practical information from gene co-expression networks in plant biology. Plant and Cell Physiology.

[82] Usadel B, Obayashi T, Mutwil M, Giorgi FM, Bassel GW, Tanimoto M, Chow A, Steinhauser D, Persson S, Provart NJ (2009). Co-expression tools for plant biology: Opportunities for hypothesis generation and caveats. Plant, Cell and Environment.

[83] Steuer R, Kurths J, Daub CO, Weise J, Selbig J (2002). The mutual information: detecting and evaluating dependencies between variables. Bioinformatics.

[84] Brunel H, Gallardo-Chacón J-J, Buil A, Vallverdú M, Soria JM, Caminal P, Perera A (2010). MISS: a non-linear methodology based on mutual information for genetic association studies in both population and sib-pairs analysis. Bioinformatics.

[85] Lindlöf A, Lubovac Z (2005). Simulations of simple artificial genetic networks reveal features in the use of Relevance Networks. In Silico Biol.

[86] Mitchell SE, Delville C, Konstantopedos P, Derous D, Green CL, Han J-DJ, Wang Y, Promislow DEL, Douglas A, Chen L, Lusseau D, Speakman JR (2016). The effects of graded levels of calorie restriction: V. Impact of short term calorie and protein restriction on physical activity in the C57BL/6 mouse. Oncotarget.

[87] Speakman JR, Mitchell SE, Mazidi M (2016). Calories or Protein? The effect of dietary restriction on lifespan in rodents is explained by calories alone. Exp Gerontol.

[88] Sohal RS, Forster MJ (2014). Caloric restriction and the aging process: A critique. Free Radical Biology and Medicine.

[89] Speakman JR, Hambly C (2007). Starving for life: what animal studies can and cannot tell us about the use of caloric restriction to prolong human lifespan. J Nutr.

[90] R Core Team (2014). R: a language and environment for statistical computing. Vienna, Austria [Internet].

[91] Meyer PE, Lafitte F, Bontempi G (2008). minet: A R/Bioconductor package for inferring large transcriptional networks using mutual information. BMC Bioinformatics.

[92] Paninski L (2003). Estimation of Entropy and Mutual Information. Neural Comput.

[93] Lusseau D, Whitehead H, Gero S (2008). Incorporating uncertainty into the study of animal social networks. Anim Behav.

[94] Newman MEJ (2006). Finding community structure in networks using the eigenvectors of matrices. Phys Rev E Stat Nonlin Soft Matter Phys.

[95] Newman MEJ (2006). Modularity and community structure in networks. Proc Natl Acad Sci U S A.

