# Supplementary material for: The effects of graded levels of calorie restriction: VII. Topological rearrangement of hypothalamic aging networks
Source: Aging (Albany NY). 2016 Apr 23;8(5):917–31. doi: 10.18632/aging.100944 (PMC4931844; doi:10.18632/aging.100944)
Supplement: Supplementary file 1 [file aging-08-0917-s001.pdf]

SUPPLEMENTARTY DATA

Please browse Full Text version to see Table S1. Expression levels of aging-associated genes relative to 12h *ad libitum* intake.

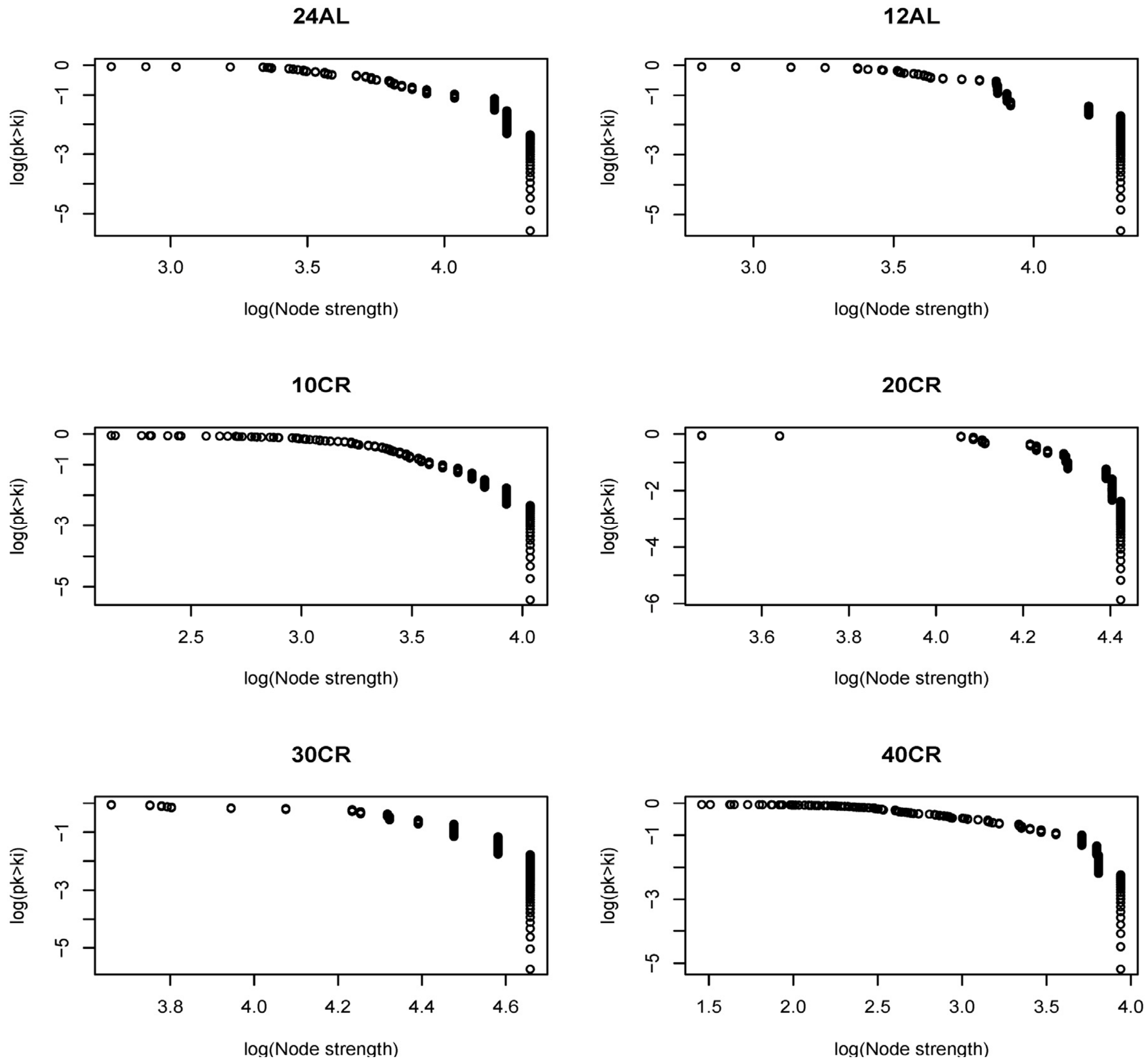

Figure S1. Node strength frequency distribution on a log-log scale. Plots assess scale free topology of the aging-associated genes networks on a log-log scale. 24AL, 12AL, 10CR, 20CR, 30CR and 40CR refer respectively to 24h *ad libitum* feeding per day, 12h *ad libitum* feeding per day, 10 %, 20 %, 30 % and 40 % restriction.

Table S2. Metrics of network topology measurements

	Clusters	Modularity coefficient	Eigenvector value (mean ± sd)	Eigenvector centrality
24AL	10	0.390	0.041 ± 0.027	0.100
12AL	10	0.397	0.035 ± 0.035	0.117
10CR	13	0.412	0.035 ± 0.035	0.128
20CR	9	0.367	0.048 ± 0.012	0.069
30CR	7	0.364	0.045 ± 0.020	0.076
40CR	12	0.441	0.030 ± 0.039	0.127
